# Recent advances in the direct cloning of large natural product biosynthetic gene clusters

**DOI:** 10.1016/j.engmic.2023.100085

**Published:** 2023-03-29

**Authors:** Jiaying Wan, Nan Ma, Hua Yuan

**Affiliations:** College of Life Sciences, Shanghai Normal University, Shanghai 200234, China

**Keywords:** Natural product, Silent *BGCs*, Genome mining, Direct cloning, Heterologous expression

## Abstract

Large-scale genome-mining analyses have revealed that microbes potentially harbor a huge reservoir of uncharacterized natural product (NP) biosynthetic gene clusters (*BGCs*), and this has spurred a renaissance of novel drug discovery. However, the majority of these *BGCs* are often poorly or not at all expressed in their native hosts under laboratory conditions, and thus are regarded as silent/orphan *BGCs*. Currently, connecting silent *BGCs* to their corresponding NPs quickly and on a large scale is particularly challenging because of the lack of universal strategies and enabling technologies. Generally, the heterologous host-based genome mining strategy is believed to be a suitable alternative to the native host-based approach for prioritization of the vast and ever-increasing number of uncharacterized *BGCs*. In the last ten years, a variety of methods have been reported for the direct cloning of *BGCs* of interest, which is the first and rate-limiting step in the heterologous expression strategy. Essentially, each method requires that the following three issues be resolved: 1) how to prepare genomic *DNA*; 2) how to digest the bilateral boundaries for release of the target *BGC*; and 3) how to assemble the *BGC* and the capture vector. Here, we summarize recent reports regarding how to directly capture a *BGC* of interest and briefly discuss the advantages and disadvantages of each method, with an emphasis on the notion that direct cloning is very beneficial for accelerating genome mining research and large-scale drug discovery.

## Graphical abstract

**Figure:**
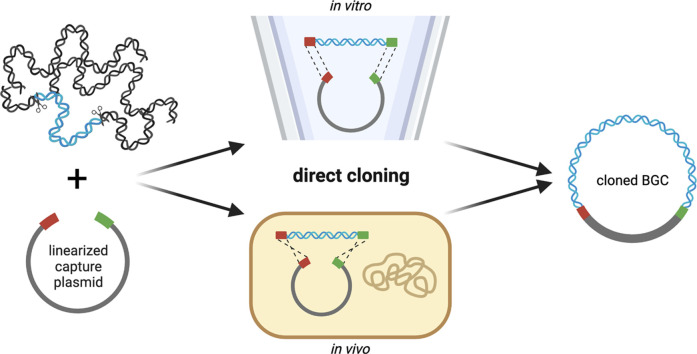

## 1 Introduction

The discovery of the Nobel Prize molecule penicillin produced by the microbe *Penicillium chrysogenum* marked the beginning of the antibiotic era, and it is regarded as a most valuable contribution to modern medicine. Since then, natural products (NPs) isolated from microbes have had a particularly large impact on human medicine, animal health, and plant crop protection. For example, numerous small molecule drugs used in the clinic are either original or modified NPs; 67% of anti-infective agents and 83% of anticancer drugs used in the past 40 years are of natural origin [1]. Most classes of antibiotics used today were identified in the 1940s to 1960s, mainly isolated through the Waksman platform-guided massive screening of microbial fermentation broths. This approach, which was very effective and fruitful in the initial screening programs, led to the creation of the Golden Age of antibiotics [2]. However, the frequency of discovery of new antibiotics from randomly screened microbes has decreased by over six orders of magnitude, from 10^−1^ to 10^−7^, with screens gradually becoming more and more unproductive due to the high rate of re-isolation or rediscovery of known compounds [3,4]. Eventually, novel antibiotic scaffolds became harder to identify using this bioactivity-guided screening strategy, causing fewer new antibiotic drugs to enter the market [5]. At the same time, the use and misuse of antibiotics led to the inevitable rise of antimicrobial resistance, which has become a looming global crisis [5,6]. Therefore, an alternative revolutionary strategy for novel drug discovery is required to meet the perpetual need for new antibiotics.

Biosynthetic studies of numerous microbial NPs have revealed that the genes responsible for biosynthesis, self-resistance, regulation, and transport are generally physically clustered in the genome forming a biosynthetic gene cluster (BGC) [7,8]. This evolved characteristic strongly facilitates horizontal gene transfer among microbes, which can lead to multiple microbial species producing the same NP [9]. Significantly, the complete genome sequencing of the first two *Streptomyces* chromosomes (from *Streptomyces coelicolor* and *Streptomyces avermitilis*) clearly established that, even though derived from two of the most well-studied bacterial species, these chromosomes contain numerous predicted *BGCs* with as yet unknown functions under standard culturing conditions [10], [11], [12]. Since then, with the advent of fast and relatively inexpensive genome sequencing technologies, microbial *BGCs* have been uncovered at an unprecedented rate. Currently, however, only a very small fraction of *BGCs* (about 2500 as of February 2023) have been characterized [13], and the vast majority are not connected to any known compounds. Because most of the annotated *BGCs* are poorly or not at all expressed in their native hosts under conventional culture conditions, they are designated as silent/orphan *BGCs*. These *BGCs* are believed to be a great resource for novel drug discovery. For example, through bioinformatics analyses of the product diversity of orphan assembly-line polyketide synthases (PKSs), Nivina *et al*. concluded that more than one-half of all orphan assembly lines could likely produce novel polyketide structures [14].

Currently, translating silent *BGCs* to their encoded products through genome mining quickly and on a large scale is particularly challenging because of the lack of universal strategies and enabling technologies. To date, a rich variety of methods have been reported for the activation of *BGCs*, which mainly include native host-based and heterologous host-based strategies. For genome mining in natural producers, the diverse methods mainly fall into the following two groups [15], [16], [17], [18], [19], [20], [21], [22]: 1) *BGC*-targeted (pathway-specific) approaches, such as the overexpression of positive regulator genes and/or the deletion of negative regulator genes, and the insertion of a strong promoter upstream of a target *BGC*; and 2) non-targeted (pleiotropic) approaches, such as ribosome engineering, media optimization, co-culturing, and the addition of elicitors. One of the advantages of this native host-based strategy is the maintenance of the integrity of the target *BGC* and the precursor supply. The genetic manipulation system developed in natural producers can facilitate the rewiring of the transcriptional regulatory circuit of the *BGC* of interest. However, when taking into consideration that developing genetic manipulation tools is varied from strain to strain, time-consuming, and often fruitless, the native host-based strategy is not generally applicable for accessing the NP-producing potential of most microbes. Moreover, this strategy is completely unsuitable for the exploitation of some genetically intractable strains and uncultivated microbes, which also possess a vast array of NP *BGCs* as revealed by metagenomics analyses [23], [24], [25], [26].

The heterologous host-based strategy, on the other hand, is more flexible and can be applied to all *BGCs* from both cultivated microbes and those yet to be cultivated in the laboratory. The steps of this strategy mainly include: 1) the cloning (or refactoring) of *BGCs* from microbial genomic *DNA*; 2) heterologous expression in a genetically tractable host (such as *S. coelicolor* and *Escherichia coli*); and 3) chemical analyses and structure determination of the heterologously expressed NPs. Therefore, two types of techniques are the key drivers of bioactive NP discovery using the heterologous host-based strategy. The first one includes the various methods developed for the cloning of a *BGC* of interest, and the second is the use of modern analytical techniques, such as high-performance liquid chromatography (HPLC), high-resolution mass spectrometry (HRMS), tandem mass spectrometry (MS/MS), and nuclear magnetic resonance (NMR), which have greatly facilitated analyses and structure elucidation of organic chemical compounds [27]. To date, PCR amplification or complete *de novo DNA* synthesis of a large *BGC* (*e.g.*, >30 kb) is still impractical for most research laboratories. Therefore, the cloning of large *BGCs* is still regarded as a rate-limiting step, although various cloning methods have been developed [28], [29], [30], such as library construction, PCR-based bottom-up assembly, and direct cloning. Currently, the construction of genomic libraries (*e.g.*, cosmid/fosmid, BAC, PAC, FAC) is used to capture all classes of NP *BGCs* [31], [32], [33], [34], [35]. However, library construction requires extensive screening (1000–2000 clones), making it laborious and time-consuming. Moreover, due to its untargeted nature, the library-based cloning method is also very inefficient in prioritizing *BGCs* from a vast number of uncharacterized *BGCs*. The bottom-up assembly methods frequently need to be performed in combination with other assembly methods (*e.g.*, type IIS restriction endonuclease [36], Gibson assembly [37]), but the assembly efficiency is severely limited by the length, amount of repetitive sequences, and GC content of target *BGCs* [37]. In addition, random mutations can be easily introduced during the multiple PCR amplifications and the assembly process itself.

In stark contrast, direct cloning offers many advantages over other cloning and assembly methods [38]. In direct cloning *BGCs* of interest are directly captured from genomic *DNA*; thus, this approach can both circumvent the library construction and screening steps and minimize the introduction of random mutations by PCR amplification. Thus, direct cloning represents the most promising strategy for the *BGC* prioritization-based large-scale discovery of novel bioactive NPs. All the direct cloning methods essentially include the following three parts: 1) the preparation of microbial genomic *DNA*; 2) the digestion of genomic *DNA* for release of the target *BGC*; and 3) the assembly of the *BGC* and the capture plasmid. In this Review, we briefly summarize recent advances in the direct cloning of *BGCs* by dissecting the above three parts of each method. In the future, we believe that complete *de novo DNA* synthesis (combined with bottom-up refactoring) of *BGCs* will rapidly facilitate NP genome mining. Currently, however, we believe that direct cloning is very important for large-scale novel drug discovery to combat the growing problem of antimicrobial resistance.

## 2 *In vivo* DNA circularization between the target BGC and a capture plasmid

### 2.1 Saccharomyces cerevisiae transformation-associated recombination-based method

**Fig. 1:**
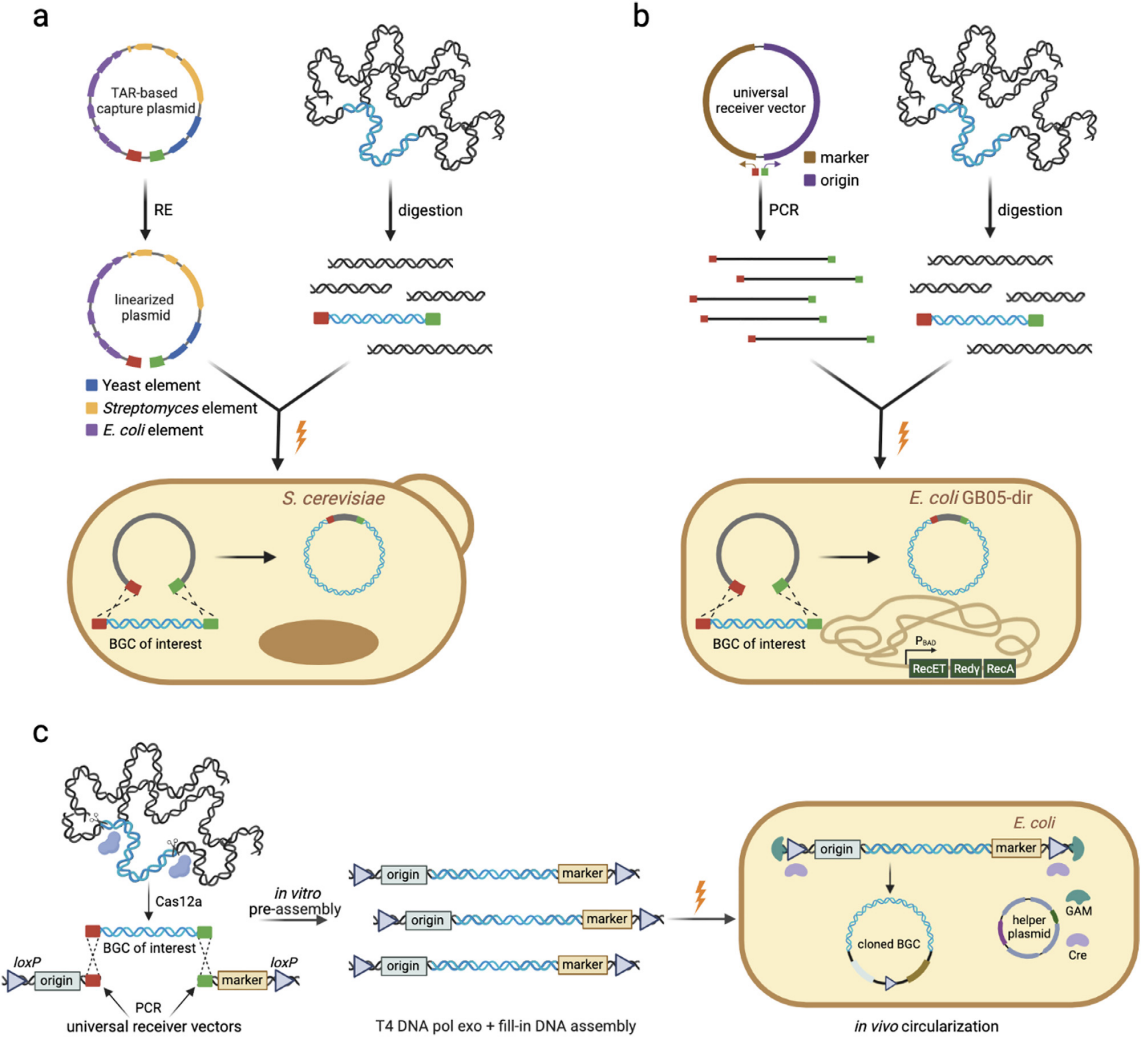
Examples of *in vivo DNA* circularization between targeted *BGC* and a capture plasmid. (a) In the *S. cerevisiae* TAR-based method, a capture plasmid with two homology arms (red and green solid boxes) is linearized via restriction enzyme (RE) digestion and co-transformed with the microbial genomic *DNA* (with/without RE digestion) into *S. cerevisiae* spheroplasts for *in vivo DNA* circularization. (b) In the LLHR method, the PCR products with two designed homology arms are obtained by the amplification of a universal receiver vector, and co-transformed with the microbial genomic *DNA* (with RE digestion) into an engineered *E. coli* strain, GB05-dir. (c) In the CAPTURE method, the capture vector is split into two fragments with a *loxP* site at the ends and obtained by the PCR amplification of designed universal receiver vectors. The target *BGC* is released by the Cas12a digestion of purified genomic *DNA* and pre-assembled *in vitro* with the two capture vector fragments via the T4 polymerase exo + fill-in *DNA* assembly approach. Finally, the pre-assembled linearized products are electrotransformed into an engineered *E. coli* strain for *in vivo DNA* circularization.

Homologous *DNA* molecules transformed into yeast are highly recombinogenic, and by taking advantage of this feature, in 1996, Larionov *et al*. developed a revolutionary method called transformation-associated recombination (TAR), which can be harnessed to selectively clone large consecutive *DNA* fragments from gently isolated human genomic *DNA* [39,40]. Fourteen years later in 2010, Kim *et al*. adapted this TAR cloning method and designed a BAC-based *S. cerevisiae*/*E. coli*/*Streptomyces* shuttle capture vector (pTARa) [26]. This pTARa vector enables direct cloning of large *BGCs* in *S. cerevisiae*, maintenance and manipulation of the cloned *BGC* in *E. coli*, and heterologous expression in *Streptomyces* hosts via integrative conjugation. In this report, it was demonstrated that pTARa could be used to both directly clone and reassemble *BGCs* of interest. The authors first employed pTARa to directly clone the 56-kb colibactin *BGC* from the sequenced *Citrobacter koseri* genome (Fig. 1a). Specifically, a capture plasmid was first constructed with one homology arm located upstream of the colibactin *BGC* and another homology arm located downstream. Then, the capture plasmid was linearized by restriction enzyme digestion and co-transformed with the *C. koseri* genomic *DNA* (without any enzymatic digestion) into *S. cerevisiae* spheroplasts for *in vivo DNA* circularization between the target *BGC* and the linear capture plasmid.

In the same report, these authors also applied pTARa to create complete *BGCs* by stitching overlapping cosmid clones. Similarly, for each reassembly experiment, a unique pathway-specific capture plasmid with homology arms was constructed. Meanwhile, the overlapping cosmids required for reassembly of a complete *BGC* were first digested with the restriction enzyme *Dra*I (which recognizes the AT-rich hexamer, TTTAAA, and mainly digests the cosmid backbone), and then co-transformed with the linearized capture plasmid into competent *S. cerevisiae* cells for the generation of a complete *BGC*. They used this strategy to successfully reassemble three complete *BGCs* (a 39-kb PKS gene cluster, an 89-kb non-ribosomal peptide synthetase [NRPS] gene cluster, and a 90-kb friulimicin BGC) from soil-derived environmental *DNA* cosmid libraries.

Later in 2014, Yamanaka *et al*. also designed and developed a *S. cerevisiae*-*E. coli*-actinobacterial chromosome integrative capture vector, pCAP01, around the SuperCos1 cosmid [41]. One of the main differences between this vector and pTARa is that the pCAP01 vector is equipped with the pUC *ori* element and thus functions at multiple copies in *E. coli*. In this report, the authors used pCAP01 to successfully TAR clone a 73-kb genomic region containing the taromycin (*tar*) *BGC* from *Saccharomonospora* sp. CNQ490, which shows sequence similarity with the *BGC* of the clinically approved antibiotic daptomycin. Specifically, the *tar* pathway-specific capture plasmid with 1-kb homologous *DNA* arms was first constructed. Then, this construct was linearized and co-transformed with the restriction enzyme *Xba*I-digested genomic *DNA* into *S. cerevisiae* VL6–48 spheroplasts. Initially, this directly cloned *tar* cluster was not efficiently expressed in the model host *S. coelicolor* M1146. However, through subsequent regulatory gene remodeling the *tar* cluster was successfully activated, leading to the production of taromycin A, which has notable structural differences from daptomycin in three amino acid residues and the lipid side chain. Remarkably, by performing *Nco*I restriction mapping analysis of two different *E. coli* clones the authors observed that the 81.8-kb pCAP01-*tar* plasmid could be stably propagated in *E. coli*. Therefore, they suggested that the pCAP01 vector can be used to directly TAR clone most NP *BGCs*.

Since the authors observed that nonhomologous end joining led to high levels of self-recircularization of the capture plasmid, which greatly reduced the efficiency of capture rates to below 2%, in 2015 they constructed another capture vector, pCAP03, with the counter-selectable marker gene *URA3* placed under the strong promoter of the *Schizosaccharomyces pombe* ADH1 gene (*pADH1*) by ligating it into the *Spe*I and *Kpn*I restriction sites of pCAP01 [42,43]. Furthermore, based on the knowledge that *pADH1* can tolerate an insertion of up to 130 bp of *DNA* between the TATA box and the transcription initiation site, the authors then designed a ready-to-use pCAP03 vector (RTU-pCAP03) for *BGC* capture. Specifically, a 144-bp *DNA* fragment with the following three features is synthesized: 1) two 18-bp overlapping fragments used for Gibson assembly with *pADH1* and *URA3* of the pCAP03 vector; 2) two 50-bp homologous *DNA* arms for recombination with *BGC* boundaries; and 3) an 8-bp *Pme*I restriction site between the two homologous *DNA* arms for linearization. Subsequently, they used RTU-pCAP03 to successfully clone the ∼22-kb thiolactomycin and 33-kb thiotetroamide *BGCs* with high efficiency (66.7% and 20%, respectively). Therefore, the RTU-pCAP03-based method can eliminate the need for PCR for capture plasmid construction and also greatly enhance the capture efficiency.

In 2019, Li *et al*. constructed pCL01 by replacing the pUC *ori* element of pCAP01 with the copy-control element from the plasmid pCC1BAC. The resulting construct pCL01 facilitates the capture of larger *BGCs* in a single-copy form, and the addition of a copy-control inducer to this construct can increase the number of cloned *BGCs* to 10–20 copies per *E. coli* cell [44]. The authors then used pCL01 to successfully capture the 5-oxomilbemycin *BGC* and enhanced the production of 5-oxomilbemycin using an advanced multiplex site-specific genome engineering strategy [44].

Notably, all the above TAR-cloned *BGCs* were introduced into *Streptomyces* hosts for heterologous expression or overexpression. To extend the number of heterologous hosts, the Moore group, in collaboration with other groups, has developed a series of TAR cloning vectors, such as the yeast/*E. coli* shuttle-*Bacillus subtilis* chromosome integrative capture vector pCAPB02 [45], and the yeast/broad-host-range Gram-negative host expression vector pCAP05 [46]. Therefore, a series of TAR cloning vectors supporting the production of NPs in diverse hosts have been developed and optimized.

### 2.2 Rac prophage RecET-based method

In 2012, Fu *et al*. developed a strategy called LLHR (linear plus linear homologous recombination) involving homologous recombination between two linear *DNA* molecules using the full-length Rac prophage protein RecE and its partner RecT [38,47]. The main steps of this strategy are as follows: 1) designing pathway-specific PCR primers and generation of a linear capture vector with two homology arms, each >30 bp; 2) restriction enzyme digestion of purified genomic *DNA* to release the *BGC* of interest; and 3) co-electrotransformation of the linear capture vector plus digested genomic *DNA* into an engineered *E. coli* strain GB05-dir (with RecET-Redγ-RecA under the control of the P_BAD_ promoter integrated in the chromosome) and validation of positive clones (Fig. 1b). They applied this platform to directly clone nine of the ten PKS-NRPS gene clusters (each 10–52 kb in length) from the *Photorhabdus luminescens* genome. Subsequently, through heterologous expression in *E. coli*, they successfully identified the metabolites luminmycin A (encoded by the *plu1881*–*plu1877* gene cluster) and luminmide A/B (encoded by *plu3263*) and proposed their biosynthetic pathways accordingly.

Of note, because of the high background levels of capture vector recircularization, the tenth and largest gene cluster, *plu2670* (∼52 kb), could not be directly cloned using LLHR alone. The authors then tried an alternative two-step, double recombination ‘fishing’ strategy by combining LLHR with Redαβ-mediated linear plus circular homologous recombination (LCHR). With this two-step cloning method, they finally obtained the 52-kb *plu2670* gene cluster (6/21 correct clones).

Later, in 2018, the same group improved the above RecET-based direct cloning method and developed the ExoCET platform (Exonuclease Combined with RecET recombination), which is based on the notion that RecET-based direct cloning efficiencies can be enhanced by annealing the linear capture vector and the targeted *BGC in vitro* prior to transformation into *E. coli* [48]. The authors used T4 polymerase as the *in vitro* 3′ exonuclease to generate 5′ sticky ends, which greatly facilitated annealing between the linear capture vector and its target BGC, thus increasing the probability of the paired *DNA* molecules simultaneously entering one *E. coli* cell. Using this optimized platform, they successfully cloned much larger *DNA* fragments (>50 kb) from bacterial and mammalian genomes with high efficiency, including the salinomycin *BGC* (∼106 kb) from both *Eco*RV- and Cas9-digested genomic *DNA* preparations. Very recently, this ExoCET platform was used to directly clone a 142-kb pseudorabies virus genome into a BAC vector [49]. The resulting infectious viral BAC was stably propagated in *E. coli*, thus facilitating studies of the biology of this virus and the development of vaccines.

Notably, in 2019, Song *et al*. used ExoCET to successfully join 12 overlapping PCR products with a linearized capture plasmid to rebuild an artificial 79-kb spinosad *BGC* in one round with a success rate of 58% [50]. In 2020, the same group developed a RedEx method (by combining Redαβ-mediated LCHR, *ccdB* counterselection, and exonuclease-mediated *in vitro* annealing) for seamless *DNA* insertion and deletion in large multimodular PKS gene clusters [51].

A number of platforms have been developed by exploiting RecET- and Redαβ-mediated homologous recombination, and these platforms have facilitated the direct cloning of *DNA* sequences from diverse and complex sources, the stitching of overlapping *DNA* fragments, and the seamless *DNA* mutation.

### 2.3 Cre-lox-based method

In 2021, Enghiad *et al*. reported a highly efficient direct cloning method named Cas12a-assisted precise targeted cloning using *in vivo* Cre-*lox* recombination (CAPTURE) [52]. The main steps of this method are as follows: 1) Cas12a-sgRNA-mediated digestion of purified genomic *DNA*; 2) PCR amplification of two pathway-specific capture plasmid fragments that each carry a *loxP* site at their ends; 3) joining the digested genomic *DNA* and the two capture plasmid fragments via the T4 polymerase exo + fill-in *DNA* assembly approach; and 4) transformation of the *in vitro* pre-assembled linearized products into an engineered *E. coli* strain (that expresses the Cre recombinase and the Redγ protein) for intramolecular *DNA* circularization (Fig. 1c). The authors applied this method to successfully clone a total of 43 uncharacterized NP *BGCs* (10–113 kb in size) from 14 *Streptomyces* and three *Bacillus* species without any failure (close to 100% cloning efficiency). Subsequent heterologous expression of the 43 clusters revealed seven *BGCs* with positive HPLC peaks, and 15 previously uncharacterized NPs were identified from five of these seven *BGCs*. Among these compounds were bipentaromycins, which showed strong antimicrobial activity toward both Gram-positive and Gram-negative bacteria. Therefore, the authors suggested that using this method to directly capture *BGCs* is inexpensive, rapid, robust, and highly efficient, and thus suitable for large-scale discovery of novel NPs.

## 3 *In vitro* DNA circularization between the target BGC and a capture plasmid

### 3.1 Gibson assembly-based method

**Fig. 2:**
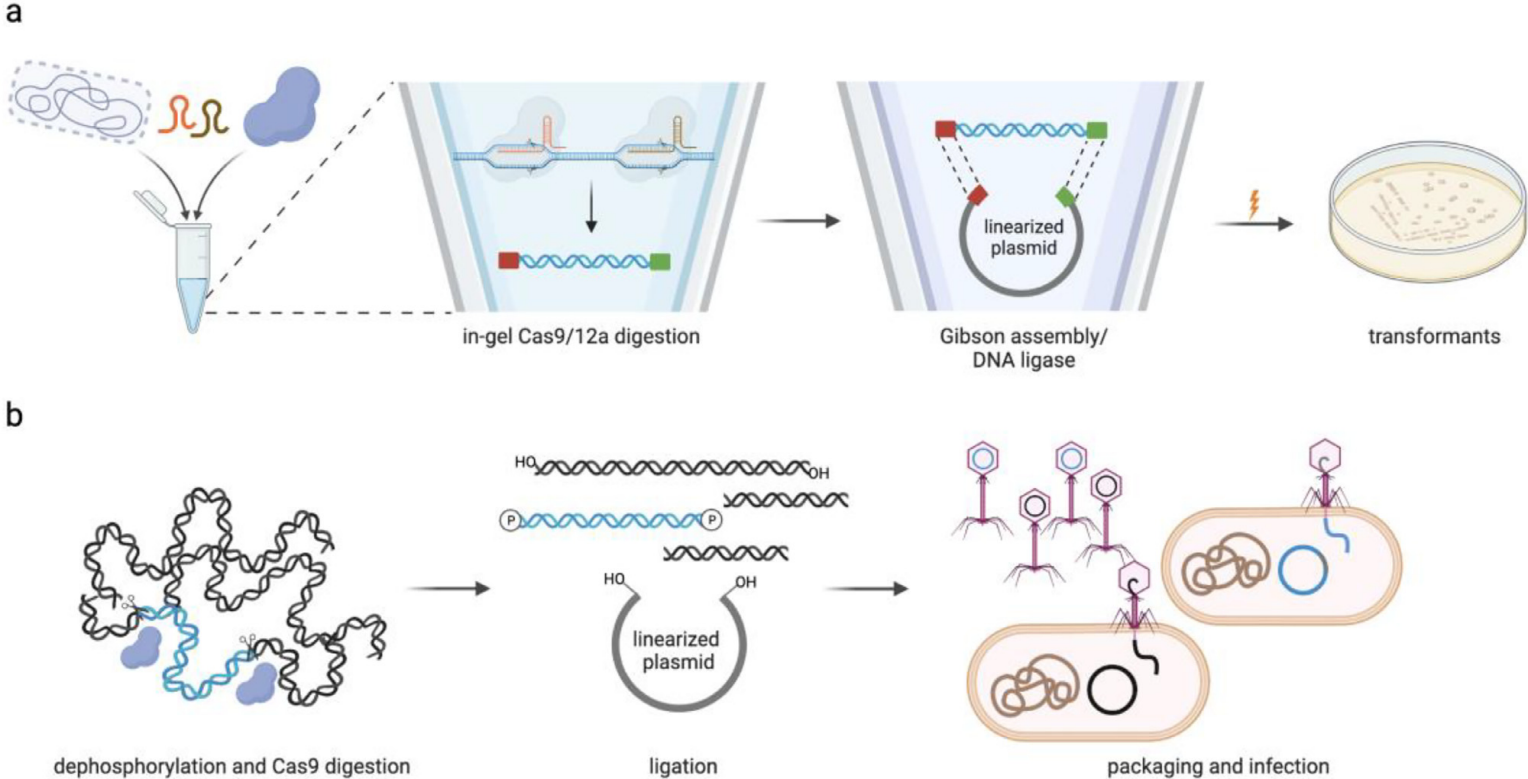
Examples of *in vitro DNA* circularization between targeted *BGC* and a capture plasmid. (a) In the CATCH or CAT-FISHING method, the microbial cells are first embedded and lysed in agarose plugs. The *BGC* of interest is released by Cas9/12a digestion, and the plugs are liquified by agarase. Then, the target *BGC* in the liquefied *DNA* mixture is used for ligation into a capture plasmid through Gibson assembly/DNA ligation. Finally, the circularization products are electrotransformed into *E. coli* cells. (b) In the *in vitro* λ packaging-assisted method, the purified genomic *DNA* is first dephosphorylated and digested by Cas9, and then ligated into the *Eco*RV-linearized and dephosphorylated universal vector pJTU2554 by T4 *DNA* ligase. The ligation products are used for the subsequent *in vitro* λ packaging and infection of E. coli EPI300.

In 2015, Jiang *et al*. reported a method, Cas9-assisted targeting of chromosome segments (CATCH), that allows the cleavage of a user-defined *DNA* region *in vitro* from intact bacterial chromosomes embedded in agarose plugs, which can be subsequently ligated with a capture plasmid through Gibson assembly [53,54]. The main steps are as follows: 1) agarose plug preparation and in-gel cell lysis; 2) sgRNA preparation and in-gel Cas9 digestion; 3) capture plasmid construction and Gibson assembly-mediated circularization; and 4) electrotransformation and validation of positive clones (Fig. 2a). As a proof of principle, the authors first demonstrated that their method could enable one-step targeted cloning of long *E. coli* genomic regions of up to 100 kb with good efficiency. Then, to demonstrate the application of CATCH, they used it to successfully clone three known NP *BGCs*, namely the 78-kb bacillaene *BGC* from *B. subtilis*, the 36-kb jadomycin *BGC* from *Streptomyces venezuelae*, and the 32-kb chlortetracycline *BGC* from *Streptomyces aureofaciens*.

Compared with traditional in-solution digestion of purified genomic DNA, the advantage of in-gel Cas9 cleavage is that the chromosomal *DNA* is well protected from mechanical shearing by the agarose gel matrix, which greatly reduces background *DNA* fragments and thus can lead to generation of fewer falsely ligated clones. In addition, the utility of Gibson assembly-mediated *in vitro* circularization saves time because the assembly products can be directly transformed into *E. coli* or other bacteria. However, when handling *DNA* segments longer than 100 kb, the CATCH method appears to be less efficient. For example, the authors obtained only one colony containg a 150-kb *E. coli* genomic fragment from three independent trials (51 colonies in total). However, when considering that most of the known microbial *BGCs* are less than 100 kb in length [13,55], this method is likely sufficient for the direct cloning of most *BGCs* of interest.

### 3.2 λ packaging-based method

In 2019, Tao *et al*. designed an elegant *in vitro* λ packaging-based method for one-step targeted cloning of NP pathways [56]. The main steps of this method are as follows: 1) genomic *DNA* isolation and dephosphorylation; 2) sgRNA preparation and Cas9 digestion; 3) T4 *DNA* ligase-mediated ligation between the digested genomic *DNA* mixture and the *Eco*RV-linearized and dephosphorylated universal vector pJTU2554 (an integrative *Streptomyces* cosmid [57]); and 4) *in vitro* λ packaging of the ligation products and infection of *E. coli* EPI300 (Fig. 2b). The authors employed this method to directly obtain the 27.4-kb Tü3010 (*stu*) and the 40.7-kb sisomicin (*sis*) *BGCs* with high cloning efficiencies of 18% and 54%, respectively. Because the λ phage has a packaging size limit of 37.4–50.4 kb (78–105% of the wild-type genome size) the cloning efficiencies for the *stu* and *sis BGCs* were also believed to test the lower and upper packaging limits, respectively. Therefore, one advantage of this method is the high λ phage packaging and infection efficiency, which will facilitate subsequent positive clone verification. Another is that the *Eco*RV-linearized vector is used for blunt-end ligation and thus can be for all pathways without the introduction of homologous *DNA* sequences for recombination. Hence, in combination with the CRISPR/Cas9 system for specific release of the target BGC, λ packaging system-assisted infection of *E. coli* cells can directly and quickly capture *BGCs* of ∼30–40 kb with high efficiency.

### 3.3 DNA ligase-based method

In 2022, Liang *et al*. reported an *in vitro* platform for directly capturing large *BGCs*, named CAT-FISHING (CRISPR/Cas12a-mediated fast direct *BGC* cloning) [55]. In this platform, the method for release of targeted *BGCs* from genomic *DNA* is similar to that of the CATCH method, but the main difference is using Cas12a instead of Cas9 for in-gel digestion (Fig. 2a). Unlike Cas9, Cas12a generates sticky ends on the target *BGC* and thus inspired the authors to develop a *DNA* ligase-based method. The capture plasmid is likewise linearized by Cas12a so as to create complementary sticky ends with the released target *BGC*. Then, the in-gel digested genomic *DNA* is ligated into the linearized capture plasmid using *E. coli DNA* ligase. Finally, the ligation products are electrotransformed into *E. coli* for validation of positive clones.

Using this CAT-FISHING method, the authors successfully cloned eight *BGCs* (with GC contents ranging from 66% to 76% and lengths from 41 kb to 145 kb) from different actinomycetes with very good efficiency (ranging from 8% to 55%). Furthermore, when combined with isolation and purification of the target *BGC DNA* fragments using pulsed field gel electrophoresis (PFGE), the capture efficiency was greatly increased to ∼70%. The high efficiency is mainly attributed to the greatly reduced number of genomic *DNA* fragments in the in-gel digested products, which reduces the interference with the ligation reaction.

## 4 Conclusions and outlook

Rapid and cost-effective sequencing technologies have led to the exponential accumulation of NP *BGCs* present in microbial genomes, the vast majority of which are uncharacterized, and thus have spurred a renaissance of novel drug discovery [58]. Because the rate of chemical decoding of silent *BGCs* cannot keep up with their identification, prioritization of *BGCs* for further activation is required. Although automated and high-throughput biofoundries have been successfully developed for discovery of new terpenoids and RiPPs (ribosomally synthesized and post-translationally modified peptides) [59,60], the lengths of the *BGCs* for such classes of NPs are relatively small and easily refactored. Currently, however, we believe that direct cloning is suitable for targeting prioritized *BGCs* from the vast number of microbial *BGCs* that have accumulated, and that it can facilitate large-scale discovery of novel bioactive NPs. As discussed above, the traditional preparation of high-quality and high-molecular-weight genomic *DNA* (especially >100 kb) may be technically challenging for most research laboratories [29]. Instead, the preparation and manipulation of genomic *DNA* in agarose gel plugs can avoid mechanical shearing and is easy to perform even though the cost is a bit high. CRISPR/Cas-mediated digestion can be designed at near-arbitrary *DNA* sites and thus is the most precise and quickest way to release a target *BGC* [53,55]. To date, the biggest *BGC* cloned using direct cloning methods is the 145-kb candicidin *BGC* (GC content 75%; PKS) from *Streptomyces albus* J1074, which was cloned by CAT-FISHING [55]. The direct cloning method is more convenient and more efficient than BAC library construction, which uses an endonuclease for partial digestion of genomic *DNA* and requires further purification of *DNA* fragments (*e.g.*, 75–145 kb) by PFGE. Notably, using BAC vectors Hashimoto *et al*. have successfully cloned the largest PKS gene cluster (for the biosynthesis of quinolidomicin, >215 kb) [35]. TAR cloning has enabled the direct cloning of a chromosomal *DNA* fragment of up to almost 300 kb in length from complex mammalian genomes, and thus so far it is undoubtedly the most reliable method for selective isolation of very large chromosomal regions [61]. However, the majority of *BGCs* of interest often contain large PKS and NRPS genes, whose intrinsically repetitive and high GC-content sequences probably lead to unexpected inter- and intra-molecular recombination and thus the formation of unwanted *BGCs* (which are difficult to detect without sequencing). In summary, although different research laboratories may leverage different methods to acquire targeted *BGCs*, coordinated efforts from academia and industry are necessarily required to provide more drug lead compounds for use in the fight against the growing problem of antimicrobial resistance.

## Declaration of Competing Interest

The authors declare that they have no known competing financial interests or personal relationships that could have appeared to influence the work reported in this paper.
